# PSG Validation of minute-to-minute scoring for sleep and wake periods in a consumer wearable device

**DOI:** 10.1371/journal.pone.0238464

**Published:** 2020-09-17

**Authors:** Joseph Cheung, Eileen B. Leary, Haoyang Lu, Jamie M. Zeitzer, Emmanuel Mignot

**Affiliations:** 1 Stanford University Center for Sleep Sciences and Medicine, Palo Alto, California, United States of America; 2 Department of Psychiatry and Behavioral Sciences, Stanford University, Stanford, California, United States of America; 3 Mental Illness Research Education and Clinical Center, VA Palo Alto Health Care System, Palo Alto, California, United States of America; University of Rome Tor Vergata, ITALY

## Abstract

**Background:**

Actigraphs are wrist-worn devices that record tri-axial accelerometry data used clinically and in research studies. The expense of research-grade actigraphs, however, limit their widespread adoption, especially in clinical settings. Tri-axial accelerometer-based consumer wearable devices have gained worldwide popularity and hold potential for a cost-effective alternative. The lack of independent validation of minute-to-minute accelerometer data with polysomnographic data or even research-grade actigraphs, as well as access to raw data has hindered the utility and acceptance of consumer-grade actigraphs.

**Methods:**

Sleep clinic patients wore a consumer-grade wearable (Huami Arc) on their non-dominant wrist while undergoing an overnight polysomnography (PSG) study. The sample was split into two, 20 in a training group and 21 in a testing group. In addition to the Arc, the testing group also wore a research-grade actigraph (Philips Actiwatch Spectrum). Sleep was scored for each 60-s epoch on both devices using the Cole-Kripke algorithm.

**Results:**

Based on analysis of our training group, Arc and PSG data were aligned best when a threshold of 10 units was used to examine the Arc data. Using this threshold value in our testing group, the Arc has an accuracy of 90.3%±4.3%, sleep sensitivity (or wake specificity) of 95.5%±3.5%, and sleep specificity (wake sensitivity) of 55.6%±22.7%. Compared to PSG, Actiwatch has an accuracy of 88.7%±4.5%, sleep sensitivity of 92.6%±5.2%, and sleep specificity of 60.5%±20.2%, comparable to that observed in the Arc.

**Conclusions:**

An optimized sleep/wake threshold value was identified for a consumer-grade wearable Arc trained by PSG data. By applying this sleep/wake threshold value for Arc generated accelerometer data, when compared to PSG, sleep and wake estimates were adequate and comparable to those generated by a clinical-grade actigraph. As with other actigraphs, sleep specificity plateaus due to limitations in distinguishing wake without movement from sleep. Further studies are needed to evaluate the Arc’s ability to differentiate between sleep and wake using other sources of data available from the Arc, such as high resolution accelerometry and photoplethysmography.

## Introduction

Sleep plays a vital role in health and well-being, yet the gold standard for measuring sleep, polysomnography (PSG) is costly and inconvenient. Over the past 25 years, collection of wrist accelerometry data (i.e., actigraphy) has proven to be a valuable contribution to the evaluation of sleep and sleep disorders clinically [1–3] as well as for research studies [4–7] by offering a low burden mechanism to monitor sleep. It can be used to monitor sleep longitudinally over weeks or even months, which would currently be infeasible with PSG. However, actigraphy does not provide the fine-grain examination of sleep states and microarchitecture provided by the PSG. Because actigraphy uses wrist movement to assess the sleep or waking state, it has difficulty differentiating quiet wakefulness (no motion) from sleep. As a result, it is known to overestimate sleep (low sleep specificity) and underestimate wake (low wake sensitivity) compared to PSG^3^. A recent review showed that clinical-grade actigraphy has an overall accuracy of 86%, sleep sensitivity was 96% and specificity was low at 33% [8].

Consumer wearables are now popular among the general public who use them for activity and sleep tracking [9,10]. Numerous brands and models are currently available with a variety of features and claims. Each brand uses a proprietary algorithm and typically do not provide access to the raw accelerometry data. In order to perform proper validation studies, comparison of minute-to-minute accelerometer vector magnitude data with PSG data is necessary. Most previous validation studies [11–14] were limited because they compared whole-night summary data (e.g., total amount of wake occurring over the course of the night) with PSG instead of comparing minute-to-minute data continuously throughout the night. As such, it was our purpose to evaluate how raw minute-to-minute accelerometry data obtained from a low-cost consumer wearable device (Arc) aligns to the clinical gold standard for determination of sleep and wake, the PSG. Specifically, in this study, we aimed to identify an optimized sleep/wake threshold value using accelerometer data for the Arc device trained by PSG data. Secondarily, we tested the accuracy of the Arc device using this optimized threshold value against PSG, as well as compared its accuracy with a clinical-grade actigraph (Actiwatch).

## Materials and methods

### Study participants

Forty-one participants were recruited at the Stanford Sleep Medicine Center on the evening of their diagnostic PSG studies. We simultaneously recruited two groups of participants—those in a training group (n = 20) and those in a test group (n = 21). Exclusion criteria are age less than 13 and individuals undergoing positive airway pressure titration sleep studies. In both groups, participants wore an Arc (Huami, Mountain View CA) on their non-dominant wrist, concomitant with a PSG evaluation of their sleep. Participants in the test group also wore an Actiwatch Spectrum (Philips, Bend OR) also on their non-dominant wrist. The study was approved by the Stanford University Institutional Review Board (IRB) and a written informed consent was obtained from all participants.

### Polysomnography (PSG)

In-lab nocturnal PSGs were performed using Somnomedics Somnoscreen Plus system (Randersacker, Germany). A standard clinical PSG montage was used, and included electroencephalography (EEG; F3/M2, F4/M1, C3/M2, C4/M1, O1/M2, O2/M1), bilateral electro-oculography (EOG), and chin electromyography (EMG). For assessment of sleep disordered breathing, oronasal thermal airflow sensor was used to monitor airflow for the identification of apnea/hypopnea events, dual thoracoabdominal belts were used for monitoring respiratory effort, continuous pulse oximetry was used for monitoring oxygen saturation. Scoring was performed by a single expert technologist using the Somnomedics Domino software (version 2.8.0). American Academy of Sleep Medicine (AASM) 2017 rules were used to determine sleep staging (wake, N1, N2, N3, and rapid eye movement [REM]) in 30 second epochs [15]. Apneas/hypopneas were scored using the AASM hypopnea criteria (flow reduction of at least 30% for at least 10 seconds followed by an arousal or a 3% oxygen desaturation) [15]. The average number of apneas/hypopneas per hour of sleep (apnea-hypopnea index [AHI]) was calculated.

### Arc and actigraph devices

Arc devices (six devices) were purchased from Huami Inc. A Samsung Android (version 7.1.1) smartphone with the Amazfit app (version 1.0.2) installed was used to communicate with the Arc devices. The app was used to synchronize data from the Arc devices before and after the PSG studies. Minute-to-minute accelerometer data were obtained from the Huami Inc’s cloud (https://github.com/huamitech/rest-api/wiki; last accessed on March 2, 2020). Actiwatch Spectrum devices (three devices) were purchased from Philips Respironics (Bend, OR). Actiwatch devices were configured to store data as the integral of activity occurring in 60 second segments. Actiwatch data were retrieved using Philips Actiware software (version 6.0.9). Time synchronization was performed across the Arc, Actiwatch, and PSG recording system the evening prior to the overnight PSG during setup.

### Data processing

Time stamps were used to align minute-to-minute raw data (vector magnitude) from the Arc device and PSG study. Arc data were cleaned by removing a series of default output values of “20” while the device was inactive. Because the PSG were scored in standard 30-second epochs while Arc and Actiwatch data were in 60-second intervals, we collapsed the PSG data into one-minute epochs. In doing so, if any 30-second epoch within the minute was scored wake, then we considered that minute as wake, allowing us to compare minute-level PSG data with Arc and Actiwatch data. In all, these split epochs (i.e., 60 s included scored W and S) only occurred in 5% of all epochs.

### Identification of wake threshold value

The core of the Cole-Kripke algorithm (Eq 1) is that time in bed is assumed to be sleep except when the activity data is of sufficient length and/or amplitude (i.e., above a Wake Threshold Value) to infer that the participant is more likely to be awake than asleep [16,17].

**Eq 1**: Total Activity = E0+(E1x0.2)+(E-1*0x.2)+(E2x0.04)+(E-2x0.04) such that E0 is the activity level in the one-minute epoch of interest, E1 is one minute later and E-1 is one minute earlier, and so on. If the Total Activity in a given one-minute epoch is less than or equal to the Wake Threshold Value, the epoch is scored as sleep (positive test). If the Total Activity in a given one-minute epoch is greater than the Wake Threshold Value, the epoch is scored as wake (negative test).

In the training group dataset, we explored a range of thresholds (2–25 units) to select a value that best aligned Arc-determined sleep/wake with PSG-determined sleep/wake (method previously described in Cheung et al. 2018 [18]).

Actiwatch data were converted into sleep and wake using algorithms built into Actiware, using each of the “auto”, “low”, “medium” and “high” settings.

### Statistical analysis

Descriptive statistics and frequency distributions were performed on all demographic and clinical characteristics. Data were evaluated for extreme or implausible values, non-normal distributions, and missingness. Summary measures are reported as means and standard deviation for continuous variables or counts and proportions for categorical, or as median and interquartile range when the data was not normally distributed. Data were split into a training and test group to identify an optimal wake threshold. The test group was used for all final performance measures. Students-t test and Chi-square tests were used to evaluate sample mean differences. Non-parametric alternatives were the Kruskal-Wallis and Fisher’s Exact. All statistical analysis was performed using R Studio Version 1.1.463.

Accuracy, sensitivity, and specificity were measures of agreement used to assess the minute-to-minute concordance between the Arc device and the PSG. Standard deviation for each measure is reported when available. Sleep or wake as scored by the PSG is set as the ground truth, with a “true positive” (TP) being when PSG-determined sleep is determined as sleep by actigraphy and a “true negative” (TN) being when PSG-determined wake is determined as wake by actigraphy. Likewise, a “false positive” (FP) was when PSG-determined wake was labeled as sleep by actigraphy and a “false negative” (FN) was when PSG-determined sleep was labeled as wake by actigraphy. Using this definition, accuracy [(TP+ TN)/total epochs], sleep sensitivity (same as wake specificity) [TP/(TP+FN)], and sleep specificity (same as wake sensitivity) [TN/(TN+FP)] were calculated. Based on prior literature, achieving sleep sensitivity of >95% and sleep specificity of >45% would be comparable to established actigraphy.

Performance [8]. We utilized a receiver operating characteristic (ROC) curve analysis to determine an optimal threshold. As we had no preconceived hypothesis concerning the weighting of sensitivity and specificity, our ROC balanced sensitivity and specificity equally.

To evaluate the possibility of systematic bias in overall sleep parameter scoring we analyzed total sleep time (TST, number of minutes of sleep during the overnight study) and wake after sleep onset (WASO, number of minutes of wake occurring after sleep onset and before the terminal awakening). The mean difference between the two measures (PSG and actigraph) was calculated to provide an estimate of bias for both TST and WASO. Bland-Altman plots [19] were used to illustrate the patterns of disagreement between the PSG and actigraph by plotting the mean difference for each individual against their mean value.

Box and whisker plots were used to compare performance measures of the Arc and the four available Actiwatch thresholds. In addition, box plots were used to show the distribution of difference between the PSG and each device/setting for TST and WASO.

## Results

Of the 41 participants, 24 were female and 17 were male, with a mean age of 42.2 ± 14.7 years (range, 19–72). Out of 41 participants, 37 were subsequently diagnosed with varying degrees of sleep disordered breathing (25 participants with AHI < 15, 14 participants with AHI 15–30, and 2 participants with AHI > 30) and 6 of whom were also diagnosed with a second sleep issue such as hypersomnia or narcolepsy. According to the PSG, participants had 478 ± 75.2 min of TST and 20.8 ± 14.0 of WASO. As can be seen in Table 1, there was no significant demographic or sleep quality differences between individuals in the training and test groups.

**Table 1:** Baseline demographic and sleep characteristics of cohort, overall and by group.

	Overall		Group 1 (Training)	Group 2 (Test)	
	(N = 41)		(N = 20)	(N = 21)	
Characteristic	Range	Mean ± SD, Median (IQR) or n (%)	Mean ± SD, Median (IQR) or n (%)	Mean ± SD, Median (IQR) or n (%)	P-Value
Age at Sleep Visit (years)	19–72	42.2 ± 14.9	40.9 ± 14.8	43.5 ± 15.2	0.58
Sex					
Male	-	17 (41.5%)	9 (45.0%)	8 (38.1%)	0.76*
Female	-	24 (58.5%)	11 (55.0%)	13 (61.9%)	
Race					
White	-	26 (63.4%)	10 (50.0%)	16 (76.2%)	0.11*
non-White	-	15 (36.6%)	10 (50.0%)	5 (23.8%)	
Body Mass Index (kg/m2)	18.4–36.1	24.5 ± 4.0	24.3 ± 4.3	24.8 ± 3.8	0.66
Primary Diagnosis					
Sleep Related Breathing Disorder Only	-	31 (75.6%)	13 (65.0%)	18 (85.7%)	0.16*
Other or Multiple Disorders Present	-	10 (24.4%)	7 (35.0%)	3 (14.3%)	
Sleep Efficiency	53.2–97.9	89.7 (9.6)	89.1 (13.8)	91.2 (8.7)	0.37*
Apnea Hypopnea Index (per hour sleep)	1.8–43.9	11.8 (11.3)	11.5 (13.5)	13.1 (10.2)	0.82*
Oxygen Desaturation Index	0.1–40.1	5.1 (8)	4.6 (8.4)	5.1 (7.6)	0.83*
PLM Index	0.0–87.9	3.3 (11.2)	1.3 (10.5)	3.6 (10.3)	0.16*
PLM with Arousal Index	0–20	0.2 (0.8)	0.1 (0.5)	0.5 (1.5)	0.16*

† = p < .05

* = non-parametric tests (Kruskal-Wallis or Fisher’s Exact)

### Training group

**Fig 1:**
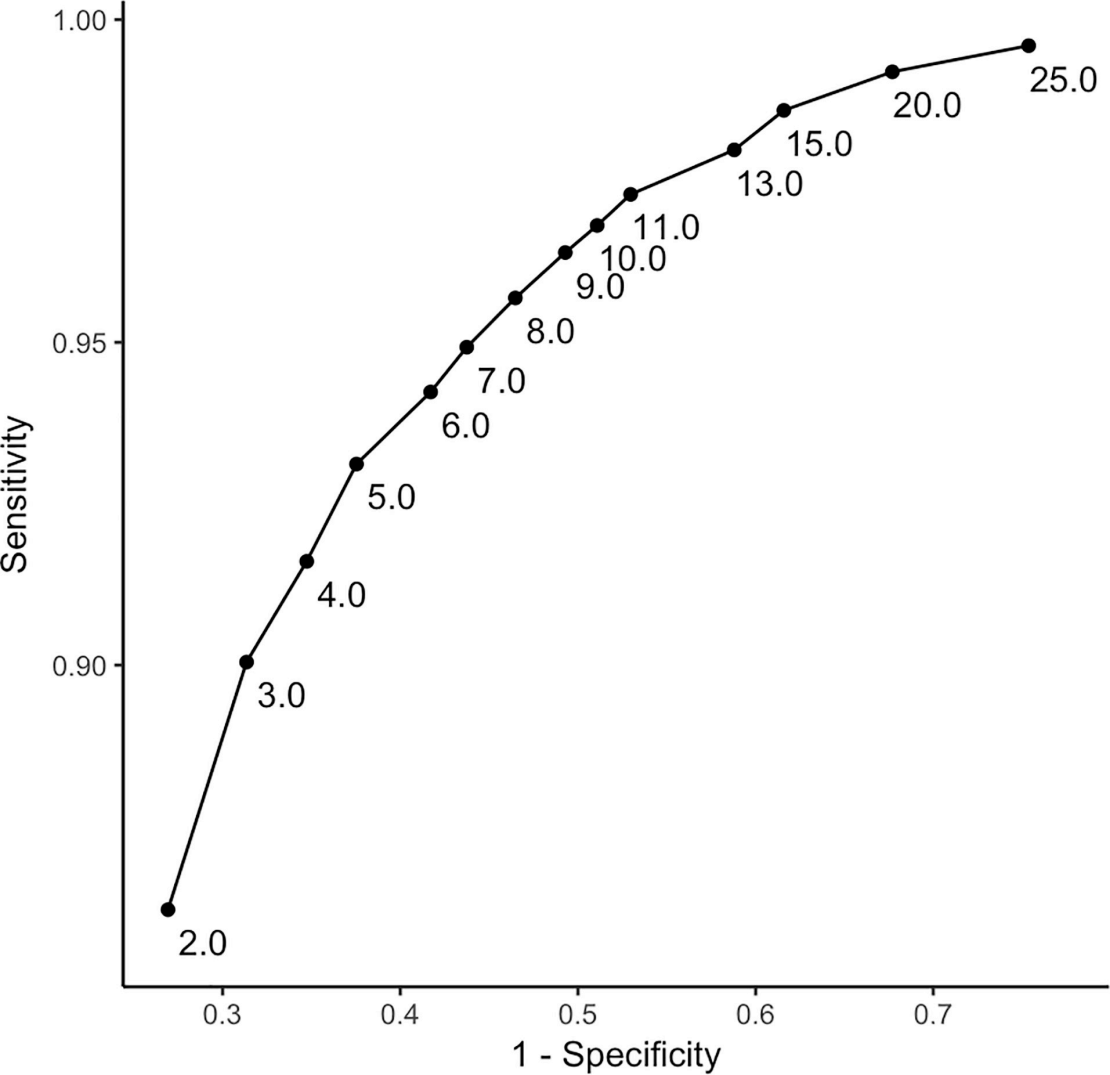
A receiver operating characteristic (ROC) curve showing sensitivity and specificity of Arc-derived sleep/wake scoring with PSG-derived sleep/wake scoring in the training group. Each point represents a different threshold.

Based on data from our training group, the ROC curve analysis indicated that a threshold of 10 units was optimal to align Arc-derived sleep/wake scoring with PSG-derived sleep-wake scoring (Fig 1). Using 10 as the threshold, comparison of the Arc to PSG resulted in an accuracy or 89.1% ±5.4%, sensitivity of 96.8% ±1.9%, and specificity of 48.9% ±17.5% (cf., Table 2).

**Table 2:** Confusion matrix of Arc sleep and wake accuracy compared to PSG in the training group using a wake threshold of 10.

		Arc			
		Sleep	Wake		
PSG	Sleep	8,140	261	8,401	Accuracy = 0.891
	Wake	831	773	1,604	Sensitivity = 0.969
		8,971	1,034	10,005	Specificity = 0.482

**Fig 2:**
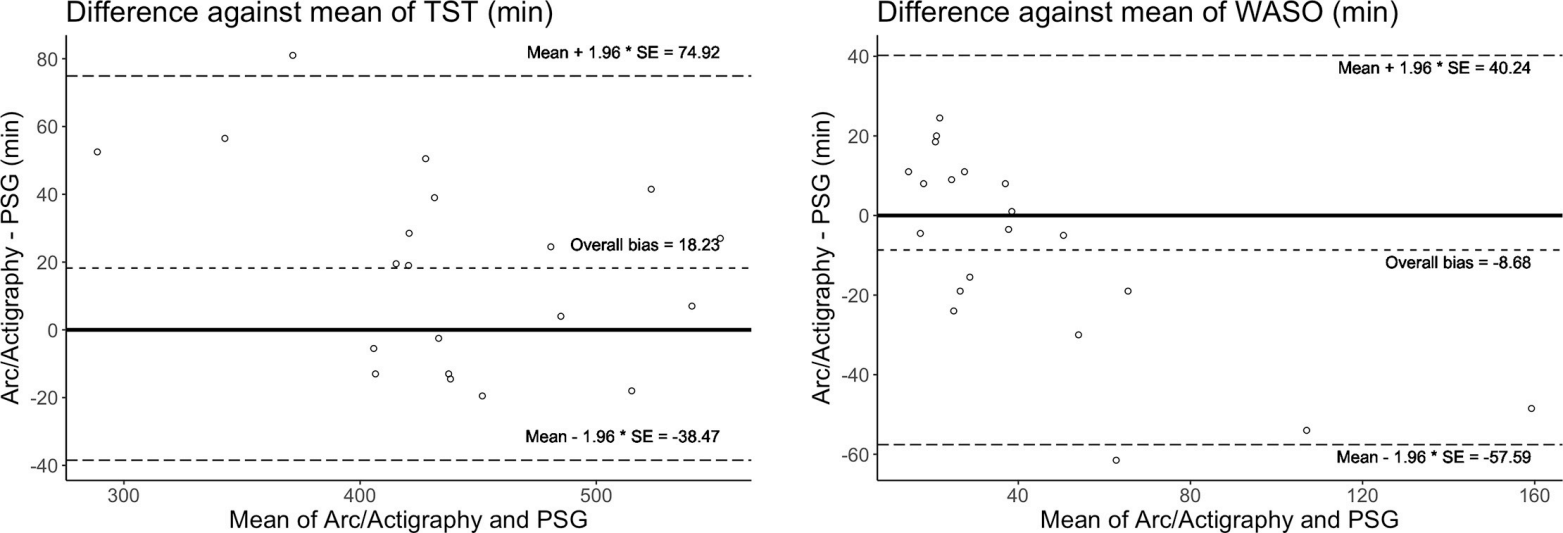
a-b. Bland Altman plots showing the difference between the Arc and PSG plotted against the mean for both total sleep time (TST) (2a) and wake after sleep onset (WASO) (2b) for each individual. Biases are marked and the dotted lines represent the upper and lower agreement limits of the biases.

A two-by-two confusion matrix using a wake threshold of 10 in Arc generated data on sleep and wake accuracy compared to PSG is shown in Table 2. The overall accuracy is 89.1%, while sleep sensitivity is 96.9% and sleep specificity is 48.2%. We also compared the performance of the Arc relative to PSG on summary statistics of sleep, notably TST and WASO. Overall, there was a bias of overestimation of TST by 18.2 ±28.9 minutes and an overall underestimation of WASO of 8.7 ±25.0 minutes, as compared to PSG. Only one participant fell outside the Bland-Altman agreement limits for each parameter (Fig 2).

### Testing group

Since the wake threshold value was optimized in the training group, we next examined whether this threshold would perform as well in a testing group. As with the training group, the threshold of 10 performed similarly well in the testing group with accuracy of 90.3% ±4.3% sleep sensitivity (or wake specificity) was 95.5% ±3.5%, and sleep specificity (wake sensitivity) was 55.6% ±22.7% (cf., Table 3).

**Table 3:** Confusion matrix of Arc sleep and wake accuracy compared to PSG in the testing group.

		Arc			
		Sleep	Wake		
PSG	Sleep	8,824	391	9,215	Accuracy = 0.904
	Wake	626	752	1,378	Sensitivity = 0.958
		9,450	1,143	10,593	Specificity = 0.546

**Fig 3:**
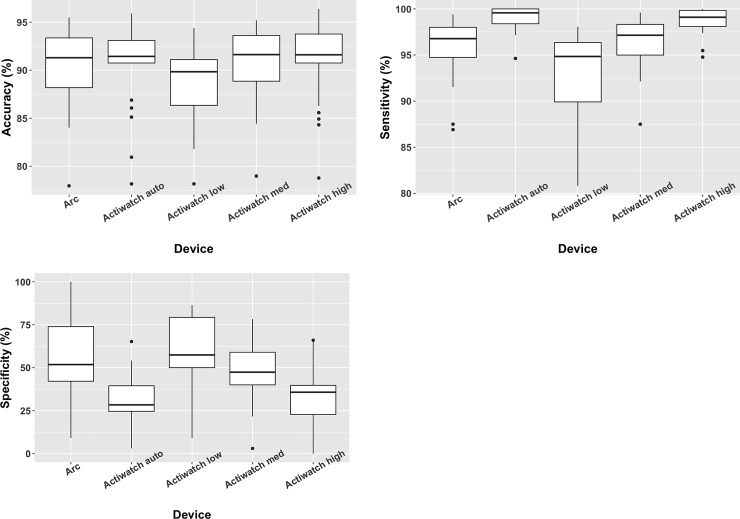
a-c: Box plots comparing the accuracy (3a), sensitivity (3b), and specificity (3c) of each actigraph device compared to the polysomnogram. Data from the Actiwatch device in the Test group was analyzed separately using all four threshold options offered by the software.

**Fig 4:**
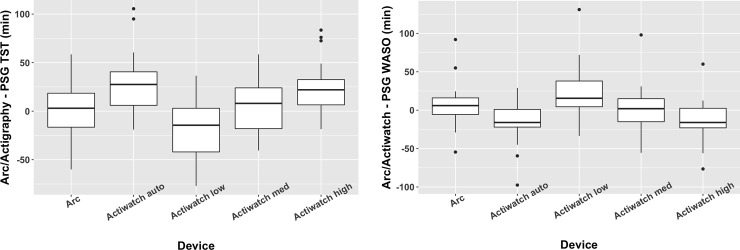
a-b: Box plots comparing the difference of each actigraph device compared to the polysomnogram for total sleep time (4a) and wake after sleep onset (4b). Data from the Actiwatch device was analyzed separately using all four threshold options offered by the manufacturer’s software.

In the testing group, we also compared the relative performance of the Arc, the Actiwatch Spectrum, and PSG. Spectrum data are typically analyzed using algorithms present in bundled software (Actiware) that rely on the user selecting one of four thresholds: low, medium, high, or auto (based on overall activity levels). We compared data derived using all four of these thresholds with data from the Arc (wake threshold = 10) and PSG. With some variance dependent on the threshold used to examine Spectrum data, comparable accuracy, sensitivity, and specificity were observed in comparing Spectrum and Arc minute-to-minute data with PSG (Fig 3A–3C). Similarly, the summary statistics of TST and WASO were comparable between the Arc and Spectrum data (Fig 4A and 4B).

## Discussion

In this study we compared minute-to-minute actigraphy raw data from the Arc device to the industry gold standard, the PSG, to evaluate the Arc’s ability to differentiate sleep and wake in sleep clinic patients. Our results suggest that Arc is comparable to an established clinical-grade actigraphy device (Philips Spectrum) in terms of epoch-by-epoch accuracy and for approximating sleep parameters. Arc weighs less than most other devices and has a prolonged battery life, collecting data for up to 20 days on a single charge, which is invaluable in a research setting. Because Arc is more than 10-fold lower in cost than FDA approved alternatives, it or similar devices could benefit many patients if it can be used more routinely in sleep consultation where it is difficult to get an accurate sleep wake history.

Arc, like all other actigraph devices, was not good at accurately determining periods of wake during sleep, having a specificity of only 54.6% in the testing group. The relatively low amount of wake that occurs during a sleep period, however, minimizes the impact of this poor differentiation on nightly summary statistics. Thus, while it is sufficient to determine summary statistics concerning sleep and wake that occur during a night of sleep, actigraphy is insufficient to replace PSG for tracking minute-to-minute changes in sleep status.

In this study, data were split into training and test datasets. Data from the training dataset was used to select an optimal wake threshold. A sleep sensitivity of >95% and sleep specificity of >45% would show the Arc is comparable to established clinical actigraphy performance [8]. Which was obtained in both the training and testing groups.

We also evaluated the Arc’s ability to estimate sleep parameters and found an overestimation of TST of 18.2 mins, and an underestimation of WASO of 8.7 mins. These findings are similar to a previous study that found that clinical actigraph underestimated WASO by 12.6 mins compared to the PSG [8].

To our knowledge, this is the first validation study where raw minute-to-minute accelerometer data (vector magnitude) from a consumer wearable device was compared to both a clinical-grade actigraph and PSG data for monitoring sleep. Our group previously investigated the performance of raw data from the Arc device against the commonly used actigraph (Actiwatch) in both a patient population (obstructive sleep apnea, disrupted sleep) and a control population. Our results showed that the Arc produced similar sleep scoring metrics compared to the Actiwatch’s auto setting with an overall accuracy of 99.0% ± 0.17%, a sleep sensitivity of 99.4% ± 0.19% and sleep specificity of 84.5% ± 1.9% [18].

However, other studies have compared processed consumer wearable data to both the PSG and clinical grade devices in varied populations including healthy adults, children and adolescents, and participants with depression or insomnia with mixed results. For instance, de Zambotti et al. [12] compared the Fitbit Charge 2 to the PSG in 44 healthy adults and found the built in algorithm overestimated TST by 9 ±24 minutes and underestimated WASO by 5 ±19 minutes, similar to our findings. However, Meltzer et al. [20] concluded that the Fitbit Ultra Normal or Sensitive sleep-recording modes did not provide clinically comparable results to PSG or clinical grade actigraphy for children and adolescents.

The strength of the current study is the use of unprocessed minute-to-minute tri-axial accelerometer data (vector magnitude) to compare to both clinical grade altigraph and PSG. By including Actiwatch in a subset of our sample, we were able to compare the performance of the Arc and the Actiwatch, a commonly used clinical grade device, against the PSG. In addition, by splitting the data into a training and test sets we were able to identify and validate an optimal wake threshold for determining the sleep/wake score for reach epoch. This allowed us to compared minute by minute data rather than just comparing final summary data (e.g. TST, WASO). Additionally, this study used clinical patients being evaluated for sleep disorders which presents a more challenging population given the likelihood for fragmented, disordered sleep.

A limitation of our study was the lack of healthy participants as the majority of our participants were subsequently found to have obstructive sleep apnea due to the nature of our sleep clinic practice. Accuracy would need to be more thoroughly evaluated in different sub-populations such as adolescents and individuals with insomnia, depression [21]. We also did not examine the possibility of inter-device variability of Arc.

There was a possibility that the epoch length of data scoring could be a problem because the PSG used 30 seconds epochs while the actigraph devices used 60 seconds. There were some 60 second periods (~5% of all data) in which the two 30 second PSG epochs were discordant for sleep (i.e., one was scored as sleep and the other as wake). In these cases we scored the epoch as wake. If we were to exclude these hybrid epochs, however, the accuracy, sensitivity, and specificity were still similar, suggesting it did not make a significant difference.

Although the data suggest that the agreement between the Arc and PSG is comparable to clinical grade actigraphy, there are some logistical issues which must be worked out before the Arc could be incorporated into a clinical practice. Currently, Arc is not an FDA-approved device and the American Academy of Sleep Medicine recommends only using FDA-approved devices when evaluating patients for sleep disorders and circadian rhythm sleep-wake disorders [22]. In addition, exporting the raw data is not a standard feature for the Arc, which makes it technically difficult to extract and analyze the raw data. Finally, as with all consumer grade devices, the hardware and firmware can be changed by the company without warning, although it is our opinion that cross validation can be quickly performed.

It is finally worthwhile to note that the Arc device studied here has the option of collecting high resolution (25 Hz) actigraphy and even photoplethysmography (PPG) data in addition to the minute-to-minute data. Higher resolution may resolve the problem of actigraphy’s low specificity. It may also have some value in detecting sleep disordered breathing or periodic leg movements during sleep. Unfortunately however, it is currently difficult to collect high resolution data for long periods of time due to the high drain on the battery. PPG measures blood volume changes in the microvascular bed of tissue by shining reflected or transmitted light on the skin which is then measured by a photosensor and such a measurement could improve actigraphy-based sleep/wake detection. [23]

## Conclusion

In this study, an optimized minute-epoch sleep/wake threshold value was identified for a consumer-grade wearable Arc using only accelerometer data trained by PSG data. With this optimized sleep/wake threshold for Arc generated accelerometer data, high minute-to-minute accuracy as well as the sensitivity and specificity findings in both groups showed the Arc’s performance was in the same class as clinical grade actigraphy when compared to the PSG. Therefore, the Arc device has great promise to be a viable low-cost option to monitor sleep-wake patterns in a sleep clinic or research environment provided the relatively low specificity (accuracy in detecting wake) is factored in when interpreting the data. This could be uniquely useful or large cohort studies. A commercial interface and FDA approval for this device would however be necessary to make this a realistic solution for clinical practice.

## Supporting information

S1 File (RAR)
